# Factors Associated with Patients Leaving Without Being Seen in a Canadian Emergency Department

**DOI:** 10.5811/westjem.47302

**Published:** 2025-12-23

**Authors:** Scott Odorizzi, Sandra Blais-Amyot, Peter Greenstreet, Omar Anjum, Jeffrey J. Perry

**Affiliations:** * University of Ottawa, Department of Emergency Medicine, Ottawa, Ontario, Canada; † The Ottawa Hospital Research Institute, Ottawa, Ontario, Canada; ‡ The Ottawa Hospital, Ottawa, Ontario, Canada

## Abstract

**Introduction:**

Patients leaving without being seen is a critical quality metric for emergency department (ED) performance and is associated with negative patient outcomes and operational inefficiencies. In this study we aimed to systematically assess patient- and system-level factors influencing leaving-without-being-seen behavior.

**Methods:**

We conducted a retrospective cohort study at The Ottawa Hospital, a tertiary-care ED with 85,000 annual ED visits in Ottawa, Canada. We analyzed all patient encounters for two years from May 2022–April 2024. Variables included demographics characteristics (age, sex), visit specifics (arrival day and time, Canadian Triage and Acuity Scale [CTAS] scores, presenting complaints), and operational metrics (ED occupancy metrics). Multivariate logistic regression analyses evaluated the influence of these factors on rates of leaving without being seen.

**Results:**

Of 170,536 ED visits, 15,473 (9.1%) patients left without being seen, and 2,716 (1.6%) left before triage. Each additional 10 years of age reduced the adjusted odds of leaving without being seen by 20.2% (older patients left less frequently). Male patients had 9.4% higher adjusted odds of leaving without being seen compared to females. For every five patients waiting to be seen, the adjusted odds of leaving increased by 16.9% for a newly arriving patient. For every five patients already seen but awaiting disposition, the adjusted odds of leaving increased by 9.6% for a newly arriving patient. Compared to CTAS 2 patients (high acuity), CTAS 3 patients had 67.1% higher adjusted odds of leaving, CTAS 4 patients had 134% higher adjusted odds, and CTAS 5 patients (lowest acuity) had 176% higher adjusted odds of leaving.

**Conclusion:**

Younger age, male sex, lower acuity, and ED crowding independently and significantly increase rates of leaving without being seen. Importantly, both crowding and volume of patients waiting impact left-without-being-seen behaviour. Optimizing patient flow through strategic movement within the ED may enhance the perception of progress, encouraging patients to remain for care.

## INTRODUCTION

Patients leaving without being seen is a key indicator of emergency department (ED) quality. High rates of leaving without being seen (LWBS) are associated with poor outcomes.^1^ Understanding the factors influencing LBWS behaviour can inform targeted interventions to improve patient care. The decision to leave without being seen can involve patient- and system-level factors. Studies show that age, sex, and acuity affect patients’ urgency perceptions and willingness to wait.^2^,^3^ Operational factors, like time of day and crowding, also influence the likelihood of leaving without being seen.^4^–^6^ Additionally, characteristics such as higher volume EDs, and being a trauma hospital or teaching center, are associated with higher LWBS rates.^7^,^8^ Understanding the relative influence of these factor can identify at-risk populations and guide strategies to reduce these rates.

In this study our goal was to assess the factors affecting patients who leave without being seen by a physician and to quantify their relative impact. We aimed to provide a comprehensive overview that can support targeted quality improvement efforts to reduce LWBS rates and improve patient safety.

## METHODS

### Study Design and Time Period

This single-center, retrospective cohort study included all ED visits from May 1, 2022–April 30, 2024. No patients were excluded. This study was granted a quality improvement exemption by the The Ottawa Hospital Research Ethics Board.

### Study Setting and Population

We conducted this study at the The Ottawa Hospital General Campus in Ottawa, Canada. It is a regional, tertiary-care and cancer center with approximately 85,000 annual ED visits. The department has 73 beds, with five beds for ambulatory fast-track patients and 24 for the remaining ambulatory patients. The ambulatory section has a secondary internal waiting room for patients after assessment to await results. The hospital uses the electronic health records system Epic (Epic Systems Corporation, Verona, WI) .

### Outcome Measures

Our primary outcome measure was the proportion of patients who leave without being seen by a physician.

### Data Collection

We collected data for all ED visits over a two-year period. A centralized database compiled information on the following: 1) demographic characteristics (age and sex); 2) visit characteristics (day and hour of arrival, Canadian Triage and Acuity Scale [CTAS] score, presenting complaint using the Canadian Emergency Department Information Systems chief complaint list, and disposition); and 3) operational characteristics (number of patients waiting to be seen and the number of patients who have been seen already but await a disposition at the time of a patient’s arrival).

### Data Analysis

We used multivariate logistic regression to assess the impact of these variables on LWBS rates. Independent variables included age, sex, day/hour of arrival, CTAS score, presenting complaint (shortlisted to the top 10 individual complaints or “other,” including all other presentations), the number of patients waiting to be seen, and those already seen but awaiting disposition at the time of patient arrival. Analyses were conducted using R v4.4 (R Foundation for Statistical Computing, Vienna, Austria).

**Population Health Research Capsule**

What do we already know about this issue?

*Patients leaving without being seen reflects ED quality and is linked to worse health outcomes*.

What was the research question?

*This study quantified how patient- and system-level factors affected the likelihood of patients leaving without being seen*.

What was the major finding of the study?

*ED crowding, younger age, male sex, and lower acuity increased these rates. An increase in the number of patients waiting raised the odds of leaving by 16.9% (OR 1.168, 95% CI, 1.161–1.177; P < .001)*.

How does this improve population health?

*Reorganization of the ED could enhance the perception of progress to make waiting more tolerable*.

## RESULTS

Of 170,536 encounters, 2,716 patients (1.6%) left prior to triage, 15,473 (9.2%) left without being seen, and 152,347 (90.8%) were seen by a physician. Among those assessed, 27,954 (18.3%) were admitted. The mean patient age was 50 years (SD 21), and 45.7% were male. The most common presenting complaints were abdominal pain (9.5%), shortness of breath (5.0%), chest pain (4.1%), generalized weakness (4.0%), and lower extremity injury (3.2%). Of those who left without being seen, the mean age was 41 years (SD 18). Despite making up 45.7% of patients, males made up 46.8% of those who left without being seen. For all patients, the most common presenting complaints amongst those leaving without being seen included abdominal pain (8.6%), chest pain (4.3%), headache (3.7%), shortness of breath (3.7%), and substance misuse/intoxication (3.7%).

The adjusted odds ratio of leaving without being seen for the top 10 most common presenting complaints are shown in the Table compared to the most common presenting complaint of abdominal pain. Age was negatively associated with leaving; each additional 10 years had a 20.2% decrease in odds (OR, 0.798, 95% CI, 0.790–0.805; *P* < .001). Males were 9.4% more likely to leave compared to females (OR, 1.094, 95% CI, 1.057–1.13; *P* = < .001). Patients arriving on Mondays and Tuesdays were less likely to leave without being seen compared to other days. Those arriving in the evening and night were more likely to leave without being seen compared to those arriving during the day. An increase of five patients in the number waiting to be seen raised the odds of leaving by 16.9% (OR, 1.169, 95% CI, 1.161–1.177; *P* < .001). An increase of five patients in the number of patients already seen but awaiting disposition raised the odds of leaving by 9.6% (OR, 1.096, 95% CI, 1.087–1.104; *P* < .001). Compared to CTAS 2 patients, CTAS 3 patients had 67.4% higher odds of leaving (OR 1.674, 95% CI, 1.590–1.763; *P* < .001), CTAS 4 patients had 134.6% higher odds (OR, 2.346, 95% CI, 2.201–2.500; *P* < .001), and CTAS 5 patients had 176.6% higher odds of leaving (OR, 2.766, 95% CI, 2.551–2.999; *P* < .001).

For those who left before triage, age was negatively associated with leaving; each additional 10 years had a 21% decrease in odds of leaving (OR, 0.79, 95% CI, 0.78–0.81, *P* < .001). Males were 9% more likely to leave compared to females (OR, 1.09, 95% CI, 1.06–1.13, P < .001). Patients arriving on Mondays and Wednesdays were less likely to leave before triage compared to other days. Patients arriving in the evening (5 pm–12 am) had 57% higher odds of leaving before triage compared to daytime arrivals (7 am–5 pm) (OR, 1.57; 95% CI, 1.45–1.71, P < .001). Those arriving overnight (12 am–7 am) posed an even greater risk, with 121% higher odds of leaving before triage (OR 2.21, 95% CI, 1.95–2.50, P < .001). An increase of five patients in the number waiting to be seen raised the odds of leaving before triage by 14% (OR 1.14, 95% CI, 1.13–1.16, P < .001). An increase of five patients in the number of patients already seen but awaiting disposition raised the odds of leaving before triage by 10% (OR 1.10, 95% CI, 1.08–1.22, P < .001).

## DISCUSSION

### Interpretation of Findings

Younger adults and patients with lower acuity presentations were more likely to leave without being seen. Men had a 9% higher adjusted odds ratio of leaving; however, they were only 0.4% more likely to leave, which is of questionable clinical significance. Arrivals on Mondays and Tuesdays had the lowest odds of leaving without being seen. Both the number of patients waiting and the number that had been seen awaiting disposition significantly increased LWBS rates. It is likely that patients perceive crowding by the number of people physically in the waiting room and by their witnessing of access block into the ED with less movement of new patients from the waiting room into the ED for assessment. Patients may be more tolerant of waits if they observe movement and less tolerant in a static waiting room.

### Comparison to Previous Studies

Our findings align with prior studies showing that men, younger adults, and low-acuity patients are more likely to leave without being seen.^3^,^8^,^9^ Lower acuity remains one of the most consistent predictors of LWBS.^9^ Our data also support a well-established link between ED crowding and higher LWBS rates.^6^ Consistent with established literature, we found a clear association between longer wait times and increased LWBS rates, emphasizing the effect of operational pressures and broader institutional issues.^10^

### Health Systems Implications

Recognizing that patients tolerate waiting better when they perceive movement,^11^ EDs should promote patient-flow strategies to possibly reduce the rate of patients leaving without being seen. Strategies like transitioning patients through different zones can create an impression of forward momentum, reducing LWBS rates without changing actual wait times.^11^ Patients who remain stationary early in their visit feel more anxious and disengaged, as they perceive they are “not in the system yet.”^12^ Designated intake or transition zones could reinforce engagement, and integrating such design and operational strategies may improve patient experience, enhance throughput, and reduce leaving without being seen rates.

Further, since acuity level remains such a strong predictor of patients leaving without being seen, having dedicated areas of the ED for low-acuity presentations may improve throughput and decrease this population’s rate of leaving. Patients arriving during overnight and early morning hours had the highest probabilities of leaving without being seen. These off-peak hours are typically associated with lower levels of physician staffing, suggesting that aligning staffing models more closely with arrival pattern data may mitigate leaving-without-being-seen behaviour by reducing wait times during high-risk periods.

### Research Implications

Further research should explore psychosocial factors influencing patients’ decisions to leave without being seen, such as perceived urgency, past healthcare experiences, access to timely primary care, and individual tolerance for waiting. Future trials testing interventions addressing these factors may offer solutions to decrease LWBS rates. The correlation between crowding and LWBS rates observed in our study emphasizes the need for further research to assess both ED-specific inefficiencies and system-wide challenges to mitigate rates of leaving without being seen and improve overall patient care. While most efforts target actual wait-time reduction, insights from queuing theory and social science suggest improving the perception of waiting may be equally valuable.^11^ Operational strategies should be investigated to improve the perception of progress of patients waiting to lower the rates of leaving without being seen.

## LIMITATIONS

This study’s comprehensive dataset from a busy, tertiary-care hospital strengthens its findings. However, as a single-center study, findings may not generalize to other settings. Local triage practices could influence presenting complaint categorization. We did not study or present any patient outcomes of the patients who left without full ED evaluation. Some factors that may influence leaving without being seen were not studied, such as arrival method. While our ED does not use an internal waiting room, EDs with such a layout may experience different patterns of LWBS behaviour than those shown in this study.

While the number of boarders may influence ED flow, our dataset only captured boarder counts at a fixed daily time point rather than dynamically at the time of each new patient’s arrival. In a univariate analysis, boarder count was moderately correlated with the number of patients already seen but awaiting disposition at the time of the new patient’s arrival (*r* 0.56). Due to this limitation and the potential for collinearity, we excluded boarder count from the final model.

## CONCLUSION

This study quantifies the impact of various factors on the behaviour of patients who leave without being seen. Patients of younger age, male sex, with lower triage acuity, and who arrived during the. evening and night had higher odds of leaving without being seen. Importantly, both the volume of patients waiting and crowding in the ED, impact LWBS behaviour negatively. Optimizing patient flow through strategic movement within the ED may enhance the perception of progress, encouraging patients to remain for care.

## Figures and Tables

**Table:** Adjusted odds of leaving without being seen by categorical factors among emergency department patients in a large, urban Canadian retrospective cohort study.

Factor	Percentage of all visits	Percentage of LWBS	Probability of LWBS	Adjusted odds ratio [95% CI]
Presenting complaint				
Headache	2.3%	3.7%	14.2%	1.80 [1.61–2.01]
Vomiting and/or nausea	2.4%	3.1%	11.4%	1.31 [1.16–1.47]
Other	59.9%	63.9%	9.6%	1.13 [1.06–1.21]
Chest Pain (cardiac features)	4.1%	4.3%	9.1%	1.83 [1.64–2.04]
Lower extremity pain	3.1%	3.0%	8.5%	1.05 [0.94–1.18]
Back pain	3.0%	2.9%	8.4%	1.19 [1.06–1.34]
Abdominal pain	9.5%	8.6%	8.0%	1.00
Upper extremity injury	2.6%	2.1%	7.1%	0.65 [0.57–0.75]
Lower extremity injury	3.2%	2.5%	7.0%	0.65 [0.58–0.74]
Shortness of breath	5.0%	3.7%	6.5%	1.00 [0.90–1.12]
Weakness – generalized	4.0%	2.2%	4.8%	0.83 [0.72–0.94]
Time of arrival				
Day (7 am – 5 pm)	58.3%	34.7%	5.4%	1.00
Evening (5 pm – 12 am)	29.2%	45.9%	14.4%	2.94 [2.82–3.05]
Night (12 am – 7 am)	12.5%	19.3%	13.9%	4.95 [4.69–5.23]
CTAS				
CTAS 1 (highest acuity)	0.7%	0.1%	1.5%	0.19 [0.11–0.32]
CTAS 2	23.0%	18.1%	7.0%	1.00
CTAS 3	55.7%	54.5%	8.7%	1.67 [1.59–1.76]
CTAS 4	13.2%	18.0%	12.2%	2.34 [2.20–2.50]
CTAS 5 (lowest acuity)	5.2%	7.8%	13.5%	2.76 [2.55–2.99]
Sex				
Female	54.1%	53.0%	8.8%	1.00
Male	45.8%	46.7%	9.2%	1.09 [1.06–1.13]

*CTAS*, Canadian Triage and Acuity Scale; *LWBS*, leaving without being seen.
